# Supplementary figures and images for: Neutrophil-mediated fibroblast-tumor cell il-6/stat-3 signaling underlies the association between neutrophil-to-lymphocyte ratio dynamics and chemotherapy response in localized pancreatic cancer: A hybrid clinical-preclinical study
Source: eLife. 2022 Sep 15;11:e78921. doi: 10.7554/eLife.78921 (PMC9512403; doi:10.7554/eLife.78921)

## Slide 1
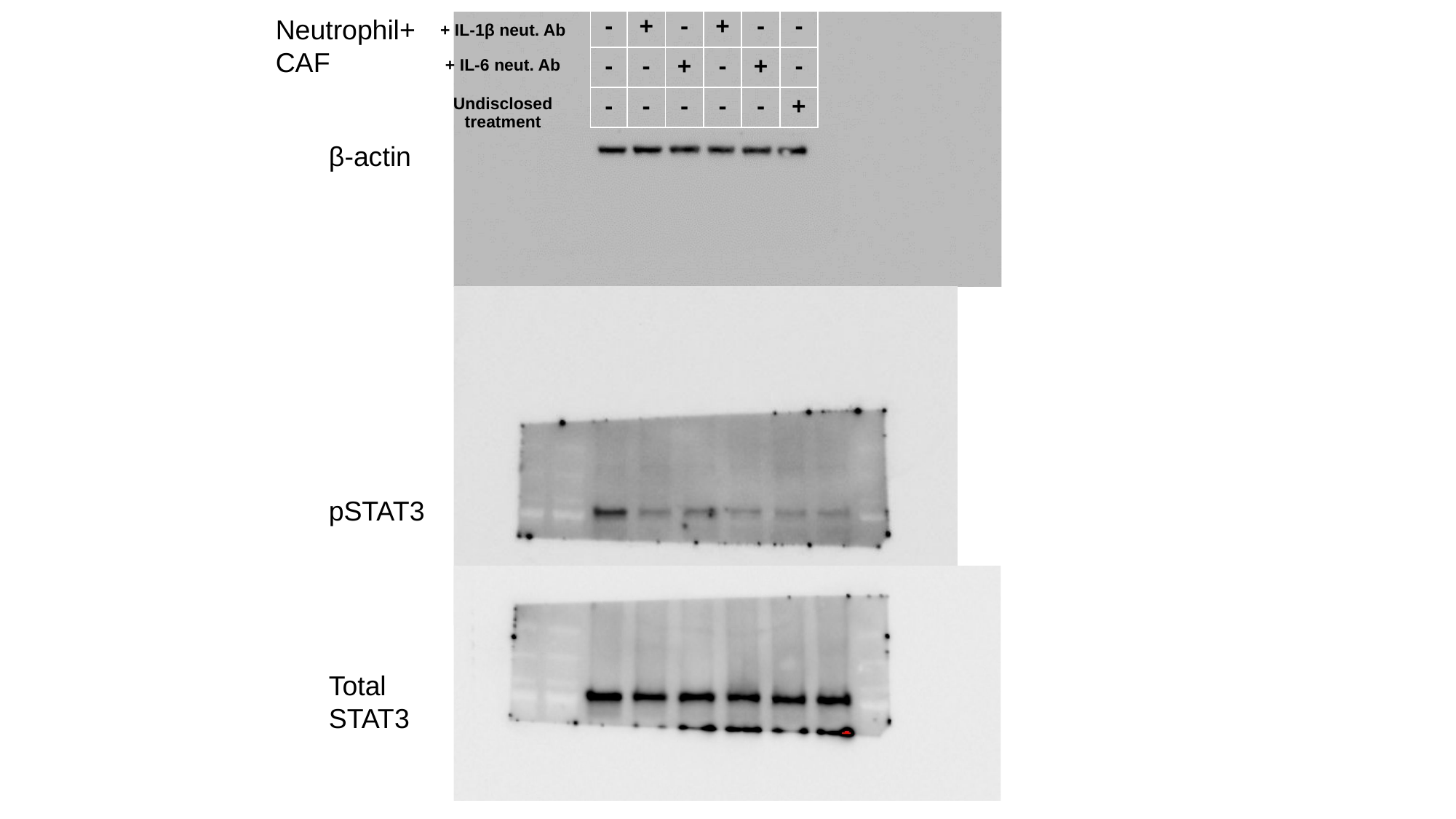

Neutrophil+CAF
| - | + | - | + | - | - |
| --- | --- | --- | --- | --- | --- |
| - | - | + | - | + | - |
| - | - | - | - | - | + |
| + IL-1β neut. Ab |
| --- |
| + IL-6 neut. Ab |
| Undisclosed treatment |
β-actin
pSTAT3
Total STAT3

Supplement: Source data 1. [file elife-78921-data1.zip › NLR chemotherapy PDAC_Source Data/Figure 5-source data 1.pptx]

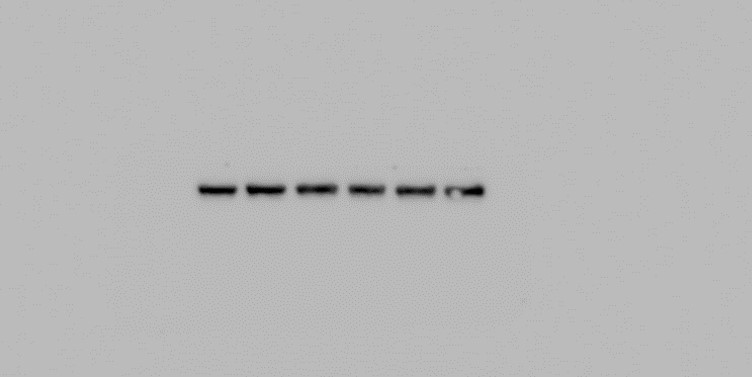

Supplement: Source data 1. [file elife-78921-data1.zip › NLR chemotherapy PDAC_Source Data/Figure 5-subfigure E-Bactin source data.jpg]

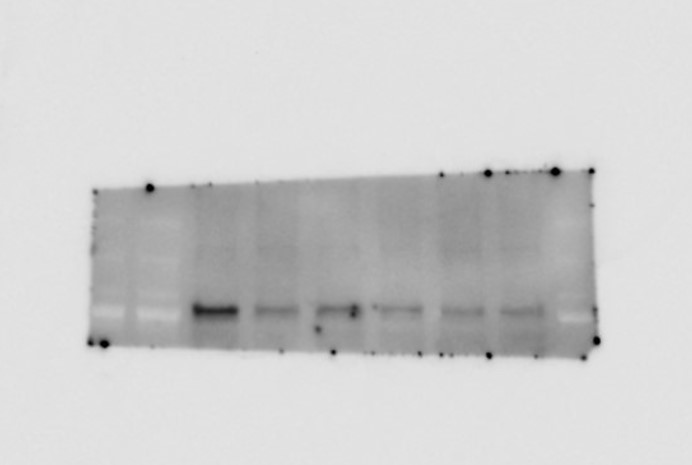

Supplement: Source data 1. [file elife-78921-data1.zip › NLR chemotherapy PDAC_Source Data/Figure 5-subfigure E-pSTAT3 source data.jpg]

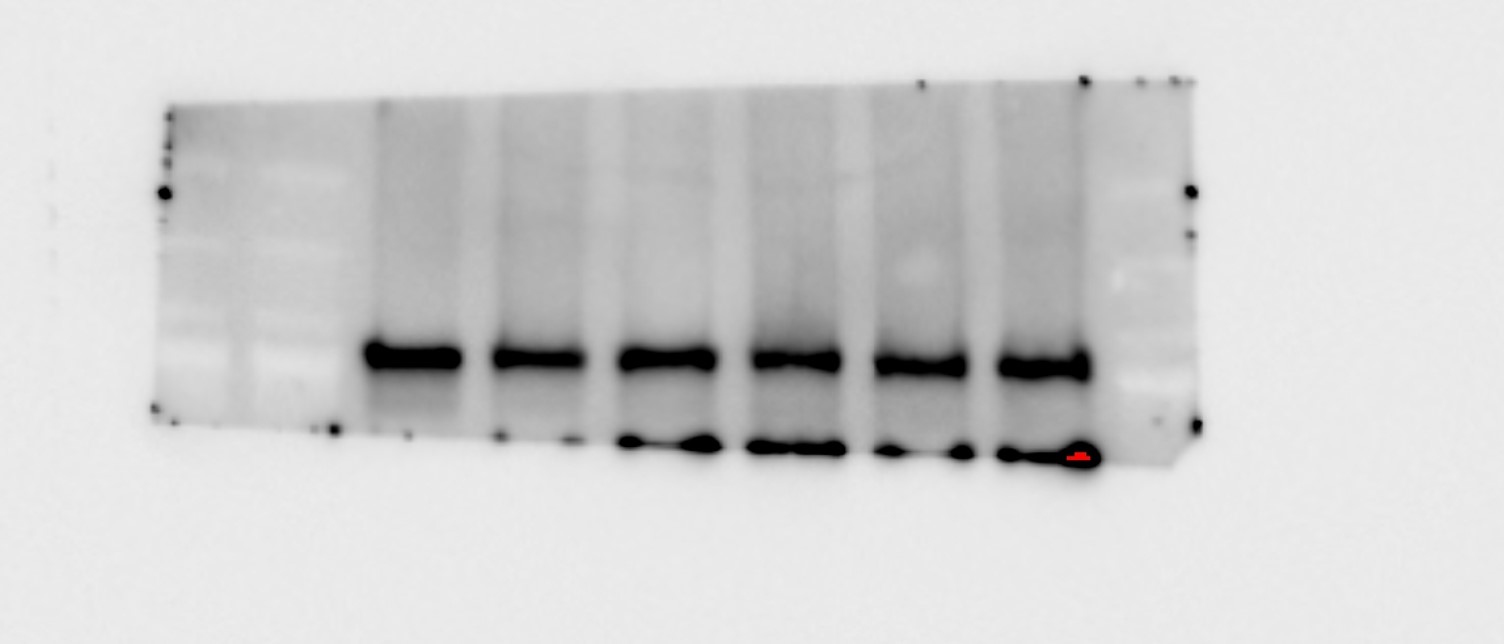

Supplement: Source data 1. [file elife-78921-data1.zip › NLR chemotherapy PDAC_Source Data/Figure 5-subfigure E-tSTAT3 source data.jpg]
